# Scaffold-Free Tubular Tissues Created by a Bio-3D Printer Undergo Remodeling and Endothelialization when Implanted in Rat Aortae

**DOI:** 10.1371/journal.pone.0136681

**Published:** 2015-09-01

**Authors:** Manabu Itoh, Koichi Nakayama, Ryo Noguchi, Keiji Kamohara, Kojirou Furukawa, Kazuyoshi Uchihashi, Shuji Toda, Jun-ichi Oyama, Koichi Node, Shigeki Morita

**Affiliations:** 1 Department of Thoracic and Cardiovascular Surgery, Faculty of Medicine, Saga University, Saga, Japan; 2 Biomedical Engineering Course Advanced Technology, Fusion Graduate School of Science and Engineering, Saga University, Saga, Japan; 3 Department of Cardiovascular Medicine, Faculty of Medicine, Saga University, Saga, Japan; 4 Department of Pathology & Microbiology, Faculty of Medicine, Saga University, Saga, Japan; North Carolina A&T State University, UNITED STATES

## Abstract

**Background:**

Small caliber vascular prostheses are not clinically available because synthetic vascular prostheses lack endothelial cells which modulate platelet activation, leukocyte adhesion, thrombosis, and the regulation of vasomotor tone by the production of vasoactive substances. We developed a novel method to create scaffold-free tubular tissue from multicellular spheroids (MCS) using a “Bio-3D printer”-based system. This system enables the creation of pre-designed three-dimensional structures using a computer controlled robotics system. With this system, we created a tubular structure and studied its biological features.

**Methods and Results:**

Using a “Bio-3D printer,” we made scaffold-free tubular tissues (inner diameter of 1.5 mm) from a total of 500 MCSs (2.5× 10^4^ cells per one MCS) composed of human umbilical vein endothelial cells (40%), human aortic smooth muscle cells (10%), and normal human dermal fibroblasts (50%). The tubular tissues were cultured in a perfusion system and implanted into the abdominal aortas of F344 nude rats. We assessed the flow by ultrasonography and performed histological examinations on the second (n = 5) and fifth (n = 5) day after implantation. All grafts were patent and remodeling of the tubular tissues (enlargement of the lumen area and thinning of the wall) was observed. A layer of endothelial cells was confirmed five days after implantation.

**Conclusions:**

The scaffold-free tubular tissues made of MCS using a Bio-3D printer underwent remodeling and endothelialization. Further studies are warranted to elucidate the underlying mechanism of endothelialization and its function, as well as the long-term results.

## Introduction

Synthetic vascular prostheses lack antithrombotic properties, biocompatibility and infection resistance. No synthetic vascular prostheses with a diameter less than 4 mm can be used in clinical settings due to early thrombotic occlusion [1–4]. Furthermore, increasing incidences of arteriosclerotic blood vessel failures, including ischemic heart disease, peripheral artery disease and other conditions, require surgical revascularization, such as coronary artery bypass grafting and lower limb bypass surgeries. The gold standard remains the usage of the patient’s own blood vessels, such as the internal thoracic artery or the greater saphenous veins. However, no synthetic vascular prostheses for the purpose of coronary artery bypass grafting or for the peripheral artery bypass to the pedal and/or posterior tibial artery are currently available [5–7]. In cases of repeat surgeries with previous surgery using multiple native vessels, surgical revascularization is not applicable due to the absence of usable native vascular conduits [8]. Similarly, vascular prostheses for the blood access in the patients on chronic hemodialysis must be refined due to frequent infection events and/or thrombosis [9,10]. A biological small caliber conduit composed of multi-cellular components without any foreign materials is thus urgently awaited. In the field of tissue engineering, an approach using a multicellular spheroid (MCS) has been considered to be promising [11, 12]. The technique utilizes the adhesive nature of the cells. When the cells were cultured in non-adhesive wells, they tended to aggregate with each other and form a spheroidal structure within 24 hours. The advantage of this approach is that multicellular components can be mixed into one spheroid, thereby promoting the formation of extracellular matrices, such as collagen or elastin. One of the coauthors (K.Na.) developed a “Bio-3D printer” system to accumulate the spheroids to create a three-dimensional structure [13]. This system enables one to create a pre-designed 3-D structure with the MCSs. With this system, tubular structures were constructed with MCSs consisting of endothelial cells, smooth muscle cells and fibroblasts. The objective of this study is to examine the feasibility and biological features of the tubular structures consisting of MCSs as an alternative small caliber vascular conduit.

## Methods

### Cell culture and the preparation of multicellular spheroids (MCSs)

Human umbilical vein endothelial cells (HUVEC), human aortic smooth muscle cells (HASMC) and human normal dermal fibroblasts (HNDFB) were purchased from Lonza, Inc. (Walkersville, MD, USA). HUVEC, HASMC and HNDFB were cultured in an appropriate medium, i.e., endothelial cell medium (ECM), smooth muscle cell medium (SMCM), fibroblast medium (FM), respectively, with growth supplement (Lonza). The cells were passaged every 3 days and were used within the third to fifth passage in this study. The cells were cultured on 0.5% gelatin-coated dishes (Techno Plastic Products, Sigma-Aldrich) and were maintained at 37°C in a humidified atmosphere containing 5% CO_2_. HUVEC were incubated with CellTracker Red (10μM) (Molecular Probes, Invitrogen Corp, Carlsbad, CA) for 30 minutes to allow visualization of the HUVEC in MCSs. Mixed cell suspensions (a total cell count of 2.5 x 10^4^) composed of HUVEC (40%), HASMC (10%), and HNDFB (50%) was plated into each well of ultra-low attachment round-shaped 96-U-well plates (Sumitomo Bakelite, Tokyo, Japan) filled with triple mixed media of ECM, SMCM, and FM with a ratio of 1:1:1. After 24 hours, the cells aggregated to form a round shaped MCS. According to the automated measurement function equipped on our Bio-3D printer, the size of the MCS was 615.0±51.3 μm (mean ±SD) for 500 MCSs.

### Bio-3D printer for generating tubular tissue made of MCSs

**Fig 1:**
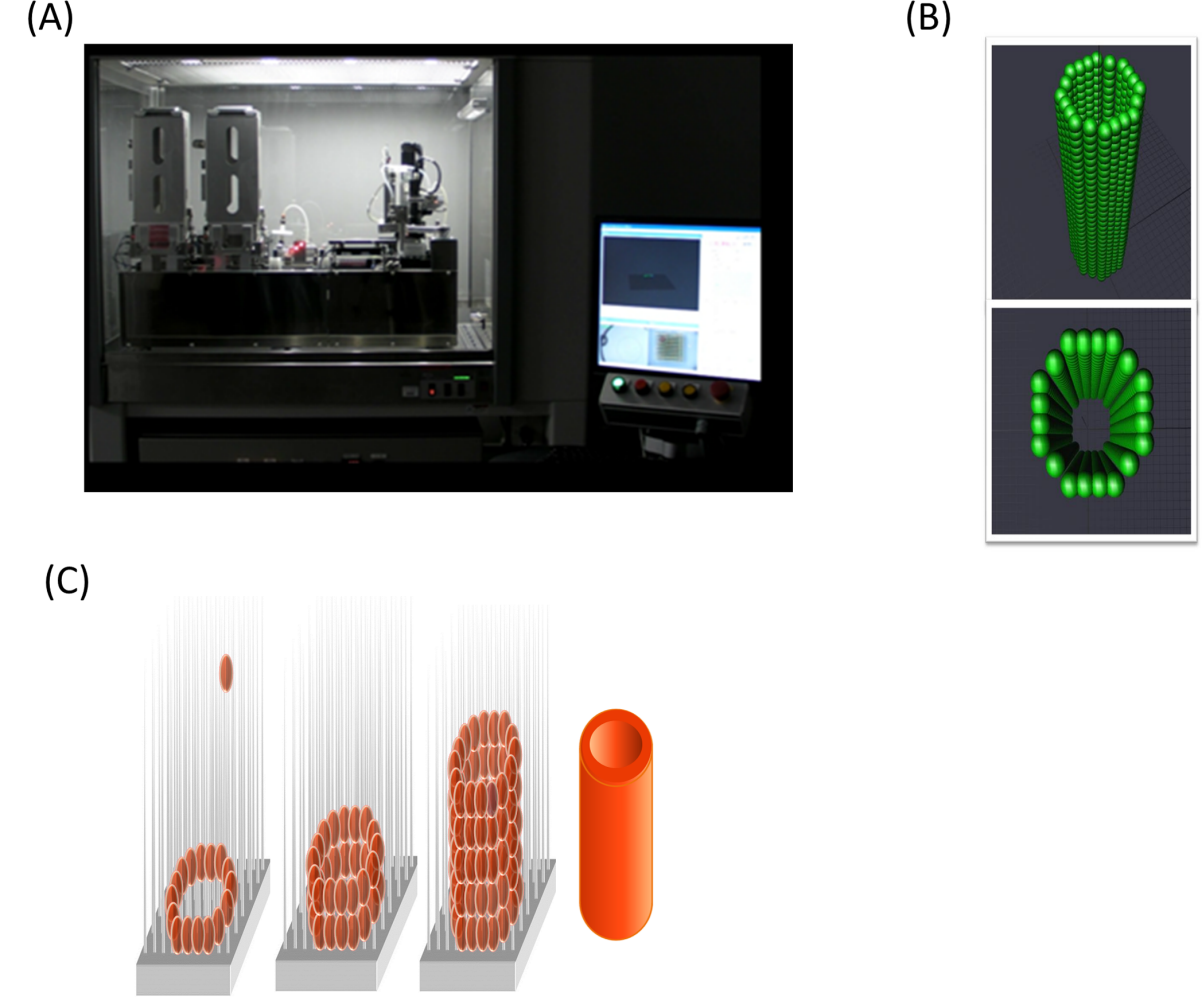
The system of lamination of the MCS (Bio-3D printer) (A) skewers the MCSs into needle-array according to a three-dimensional structure pre-designed on a computer system (C). It is possible to design a 3D tube-shaped structure, such as that plotted in green (B), on the workstation computers.

We used a “Bio-3D Printer” (Cyfuse Biomedical K.K., Japan) to assemble MCSs for constructing scaffold-free tubular tissue. According to a three-dimensional structure predesigned on a computer system, the “Bio-3D Printer” skewers MCSs into a 9 x 9 needle-array. The outer diameter of each needle was 0.17 mm, and the distance between each needle was 0.4 mm. The size of the needle array was a square, 3.2 mm in length on each side (Fig 1A and 1B, S1 Video). In this system, the MCSs were aspirated by a robotically controlled fine suction nozzle (O.D of 0.45 mm and I.D. of 0.23 mm) from the 96-well plate and inserted into the needle-array made of multiple medical-grade stainless needles. A total of 500 MCSs were created into a 3D structure robotically according to the pre-designed configuration. The time required for the placement was approximately 1.3 hours. Four days after the placement of the MCSs onto the needle-array, the needle-array was removed (Fig 1C). A duration of four days was chosen according to the results of our preliminary experiment, and the configuration of the structure was retained after the removal from the needle array due to fusion between the MCSs.

### Perfusion with a bioreactor

**Fig 2:**
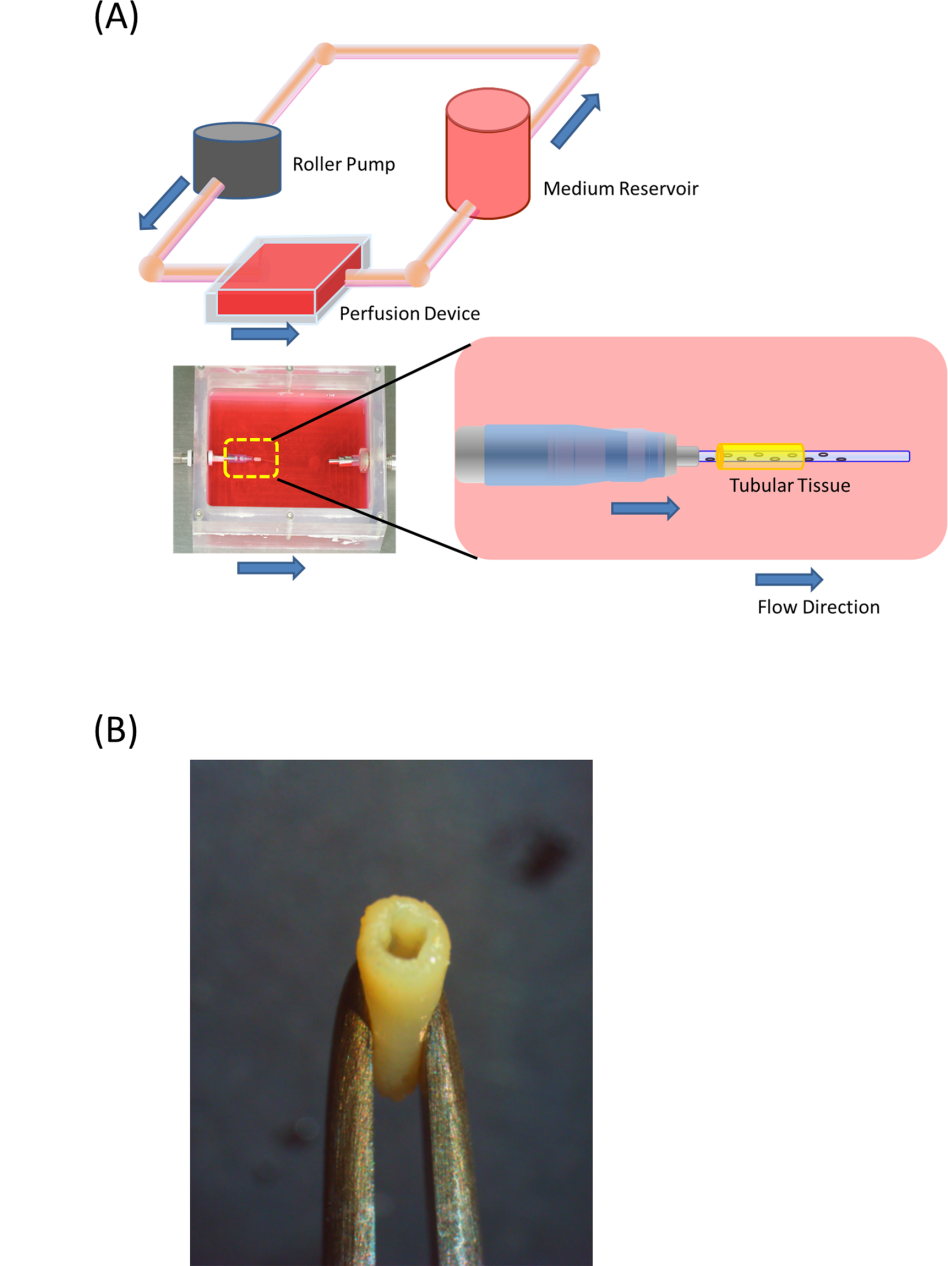
The schematic illustration of the bioreactor system (A). The vascular graft generated by the Bio-3D printer is cannulated by an outer 22 gauge intravenous catheter (SURFLO: Termo, Tokyo, Japan) which has side holes, and is perfused by culture medium for 2 days before implantation. A scaffold-free vascular graft is generated from the MCSs(B).

The above obtained tubular tissue was mounted on a bioreactor. The bioreactor has a 22 gauge plastic catheter (Terumo, Tokyo, Japan) with side holes. The catheter was placed inside the tubular tissue, and the system was perfused with the triple mixed medium of ECM, SMCM and FM for four days, first two days at a rate of 2 ml/min and the following two days at 4 ml/min (Fig 2A). A minimal flow rate was empirically chosen to avoid the destruction of any tubular tissue due to high shear stress. At this point, a tubular tissue of 1.5 mm in diameter and 7 mm in length was obtained (Fig 2B). Ideally, the flow velocity of the abdominal aorta of rats (15 to 20 cm/sec) should have been used for the bioreactor, however, we were unable to achieve this velocity due to a technical limitation.

### Implantation of tubular tissue in nude rats

**Fig 3:**
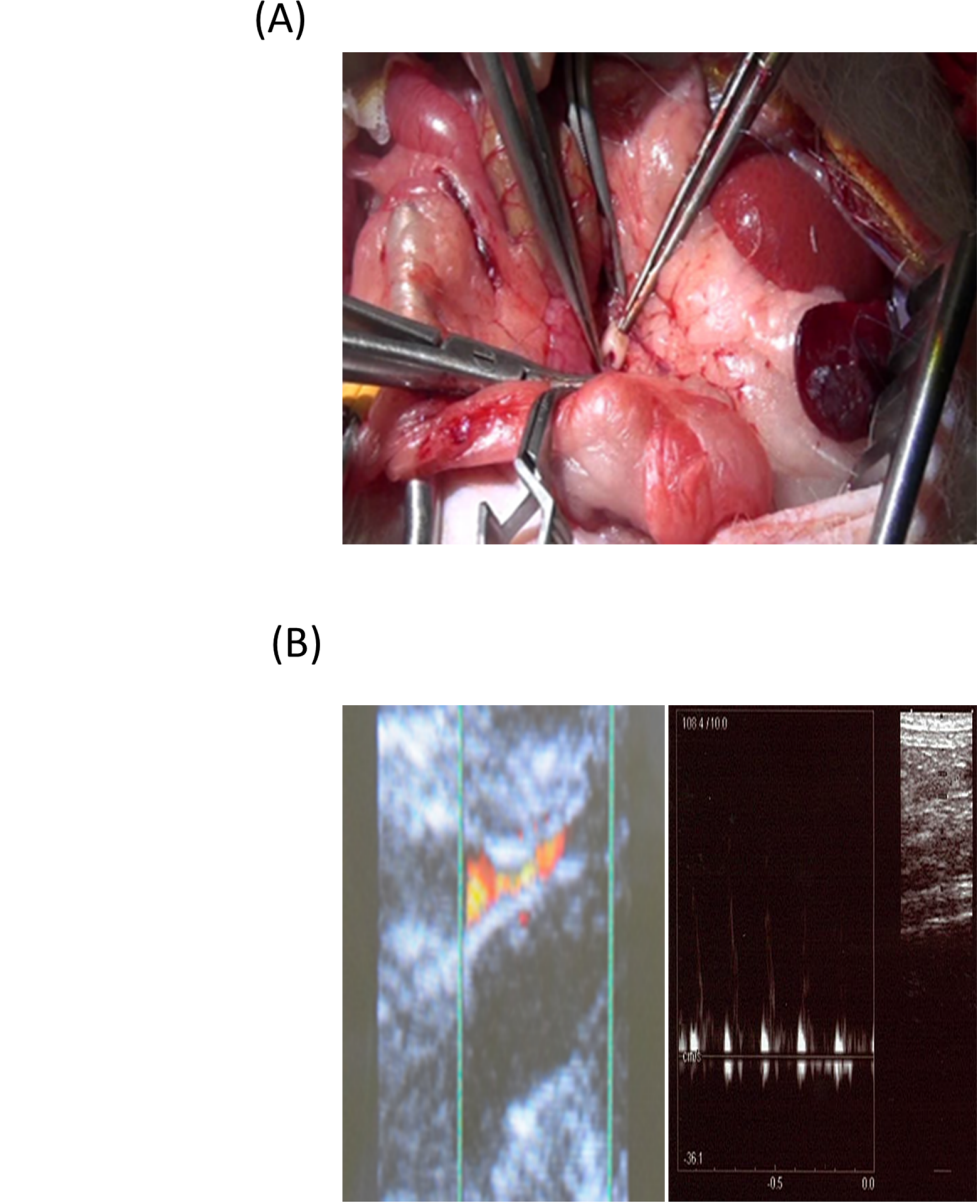
An intra-operative photograph of end-to-end anastomosis between the tubular structure and the abdominal aorta of the nude rat (A). Color Doppler (left) and pulse Doppler (right) flow imaging of percutaneous ultrasonography show patent vessels on the fifth day after implantation (B).

Fifteen male F344-rnu/rnu athymic nude rats (10-week-old, CLEA, Tokyo, Japan) were used for this experiment. We used nude rats because the cells were of human origin. Without the use of nude rats, the implantation of the vascular construct would evoke severe rejection due to the introduction of xenogeneic substances. The tubular tissue was implanted into the infrarenal abdominal aortas under anesthesia with 1.5% isoflurane (vol/vol air). The end-to-end anastomosis was performed with a 9–0 polypropylene continuous suture (Fig 3A). No anticoagulation or anti-platelet drugs were given. The flow in the graft was assessed by ultrasonography (Toshiba, Tokyo, Japan). An off-line measurement of the average flow velocity was performed from the photocopied velocity wave forms using the Image J software program (National Institutes of Health, USA). At the end of the experiment, laparotomy was performed and both ends of the tubular tissue were clamped and the tubular tissue was removed. The animals were euthanized using the overinhalation of isoflurane.

### Animal care

This study was carried out in strict accordance with the recommendations in the Guide for the Care and Use of Laboratory Animals of the National Institutes of Health. The protocol was approved by the Institutional Animal Care and Use Committee of Saga University (Approval number 25-009-1). All efforts were made to minimize suffering.

### Histological and immunohistochemical examination

Histological examinations were performed on the second (n = 5) and fifth day (n = 5) after transplantation The spheroids were fixed with 10% neutral buffered formalin and embedded in paraffin before undergoing “3D-Printing” and constructed into tubular tissues before and after implantation (on the second day and the fifth day). We performed a morphological analysis of the MCSs and the tubular structure on hematoxylin-eosin (HE) stained and Masson’s trichrome stained sections by light microscopy. To detect HUVEC, we performed an immunohistochemistry analysis for endothelial cell markers von Willebrand Factor (vWF) and CD31. For HASMC, immunohistochemical staining was performed for α-smooth muscle actin (SMA) and desmin. Deparaffinized sections were immunostained by the avidin-biotin complex immunoperoxidase (ABC) method. Furthermore, we analyzed the distribution of CellTracker Red-labeled HUVEC by fluorescent microscopy. With this staining, the number of endothelial cells lining along the lumen was counted and normalized to the luminal circumference (per millimeter). For comparison purposes, a similar endothelial cell count was performed for native rat aortae (n = 5) stained with CD31. The lumen area and the wall area at the short axis cross-section of the vessel were measured both before and after transplantation (Keyence, Japan)

### Statistical Analysis

Statistical analyses were performed using the IBM SPSS Statistics software program (version 21). Data are expressed as the mean±SD. Comparisons between the two groups were performed with the use of the Mann-Whitney U test. A probability value of <0.05 was considered to be significant.

## Results

### Patency of the tubular tissue

The implanted tubular tissues were all patent at the end of the study. We confirmed the blood flow by percutaneous ultrasonography in all cases on the second (n = 5) and fifth day (n = 5) (Fig 3B). The average flow velocity before and 2 and 5 days after implantation showed no significant differences (17.2±2.9, 16.9±7.4, 15.0±10.2 cm/sec, respectively). In all cases, a necropsy revealed no thrombosis of the tubular tissue.

### Histological examination of MCSs and the tubular tissue

**Fig 4:**
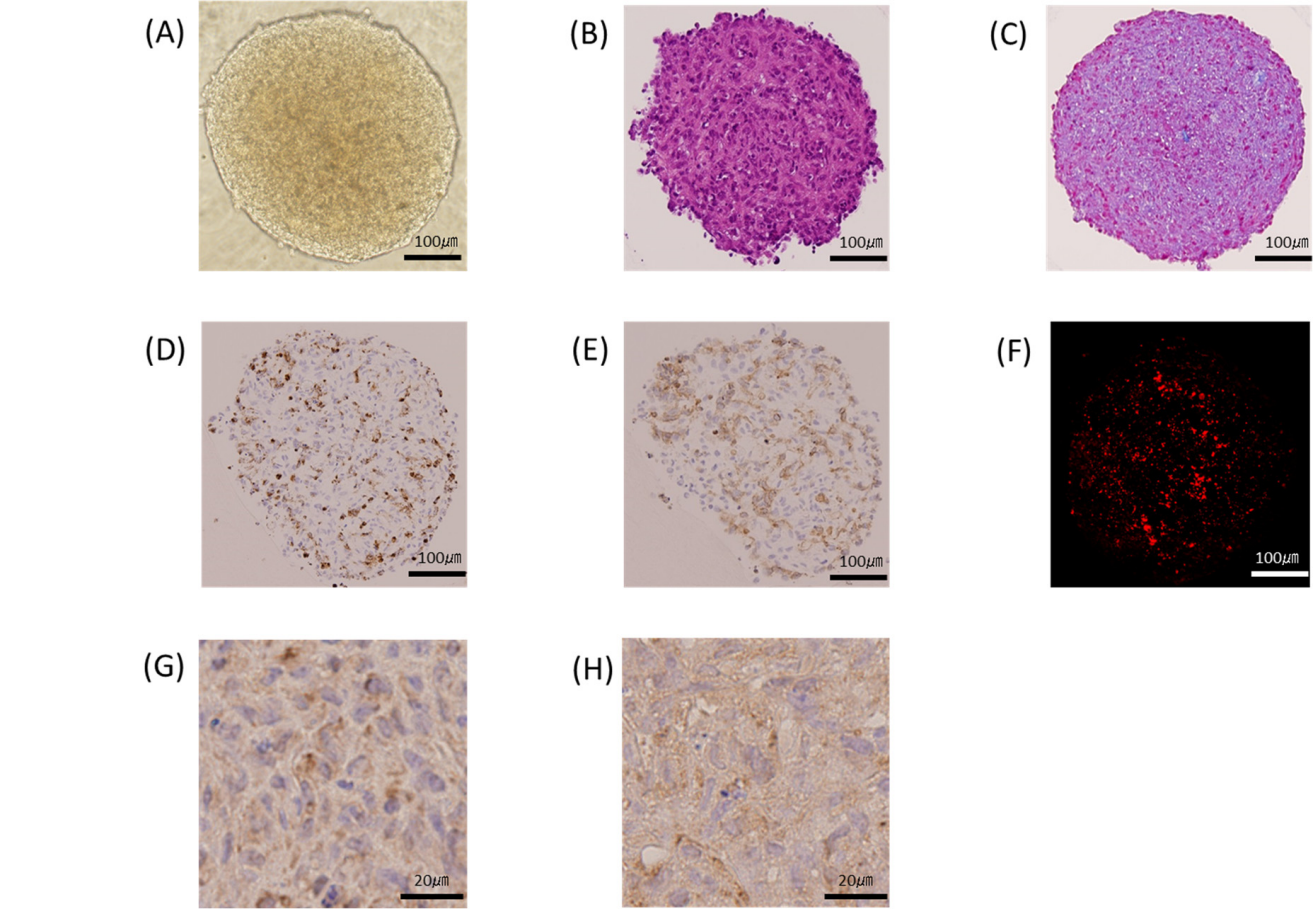
A phase contrast microscopy shows the spheroid morphology of a MCS (A). Spindle to polygonal cells are mixed in the MSC (B). Masson’s trichrome staining reveals the extensive collagenous extracellular matrix (ECM) as blue (C). vWF, CD31 and CellTracker Red-positive vascular endothelial cells are distributed to all parts of the MCS (D-F). SMA and desmin-positive HASMC intermingle with the other cell types (G,H).

A phase contrast microscopy demonstrates the spheroid morphology of a MCS (Fig 4A). Spindle to polygonal cells were mixed in the MSC (Fig 4B). Masson’s trichrome staining revealed the extensive collagenous extracellular matrix (ECM) as blue (Fig 4C). vWF, CD31 and CellTracker Red-positive vascular endothelial cells were distributed to all part of the MCS (Fig 4D–4F). SMA and desmin-positive HASMC intermingled with the other cell types (Fig 4G and 4H).

**Fig 5:**
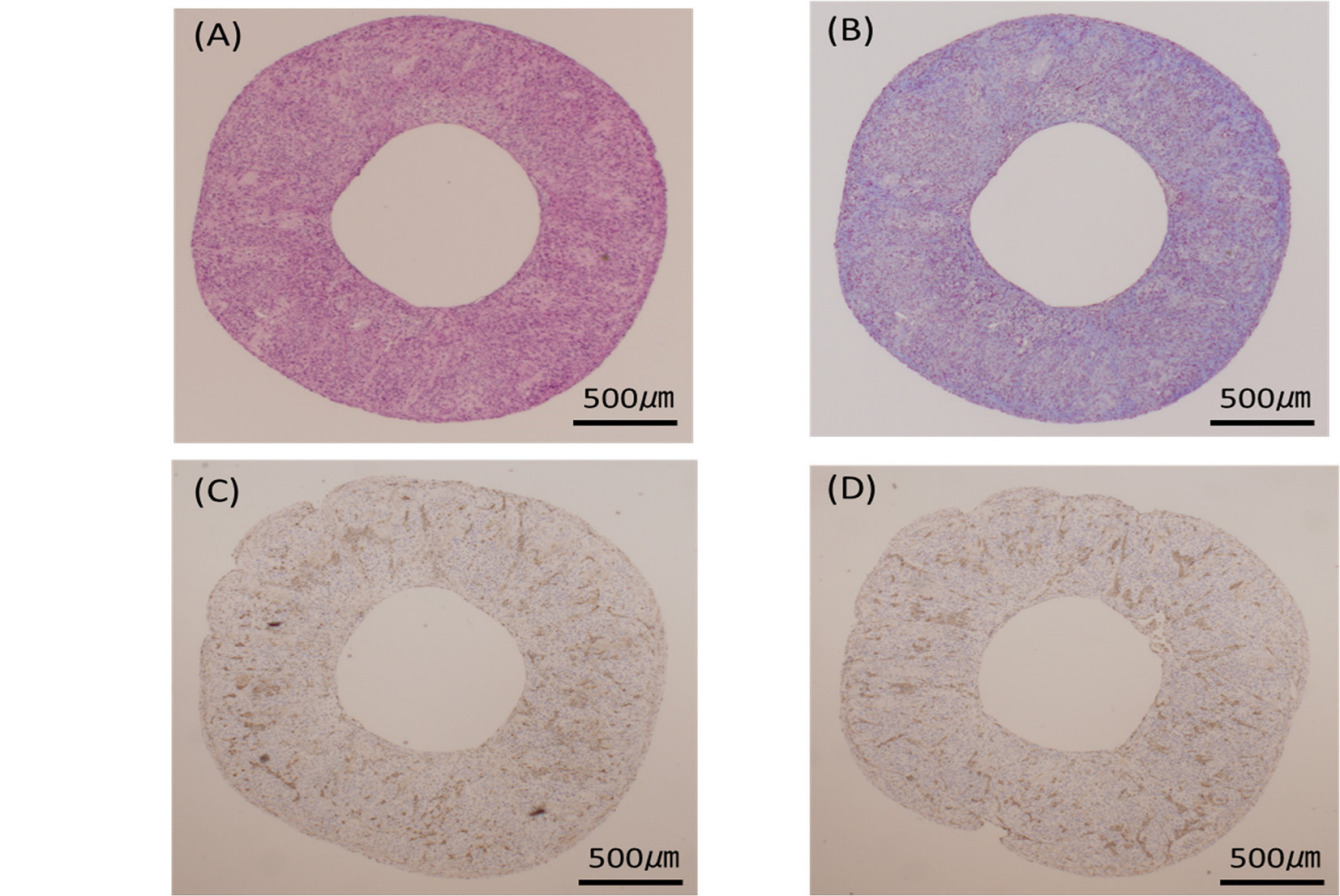
Histological examination of the vascular graft in a short axis cross-section (Pre-implantation). Hematoxylin and eosin staining reveals the internal and external margins are smooth and the pinhole by the pinholder are completely closed (A). Masson’s trichrome staining reveals the extensive collagenous ECM as blue (B). vWF (C) and CD31-positive vascular endothelial cells (D) are distributed to all parts of the graft.

**Fig 6:**
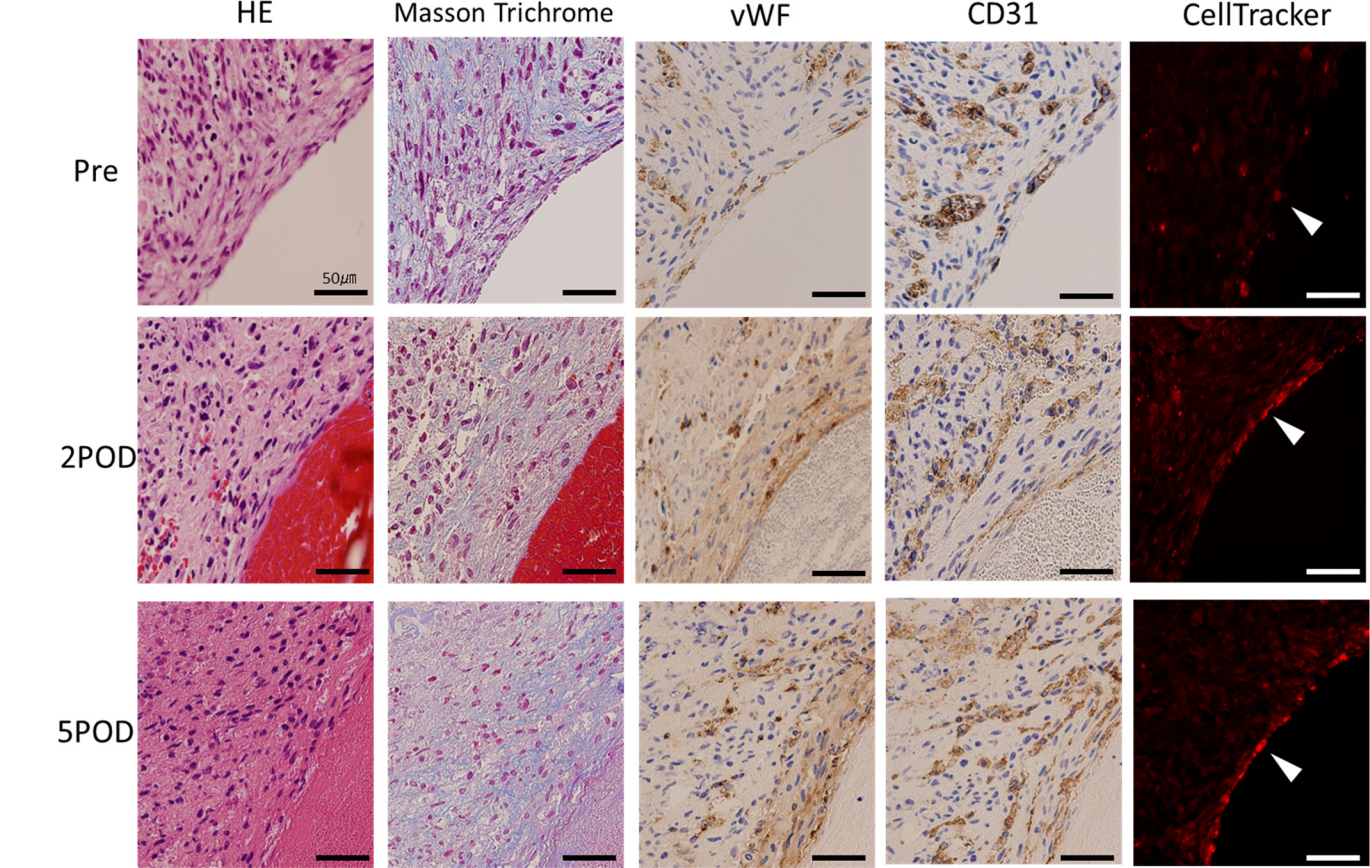
Remodeling of the blood vessel (Post-implantation). The graft of post-implantation is patent and remodeled (A). The wall area and lumen area pre-implantation are shown in blue, post-implantation in red (B). The lumen area is enlarged (P = 0.032) and the wall area is decreased (P = 0.008) after implantation. The total wall area and lumen area shows no significant difference (C).

**Fig 7:**
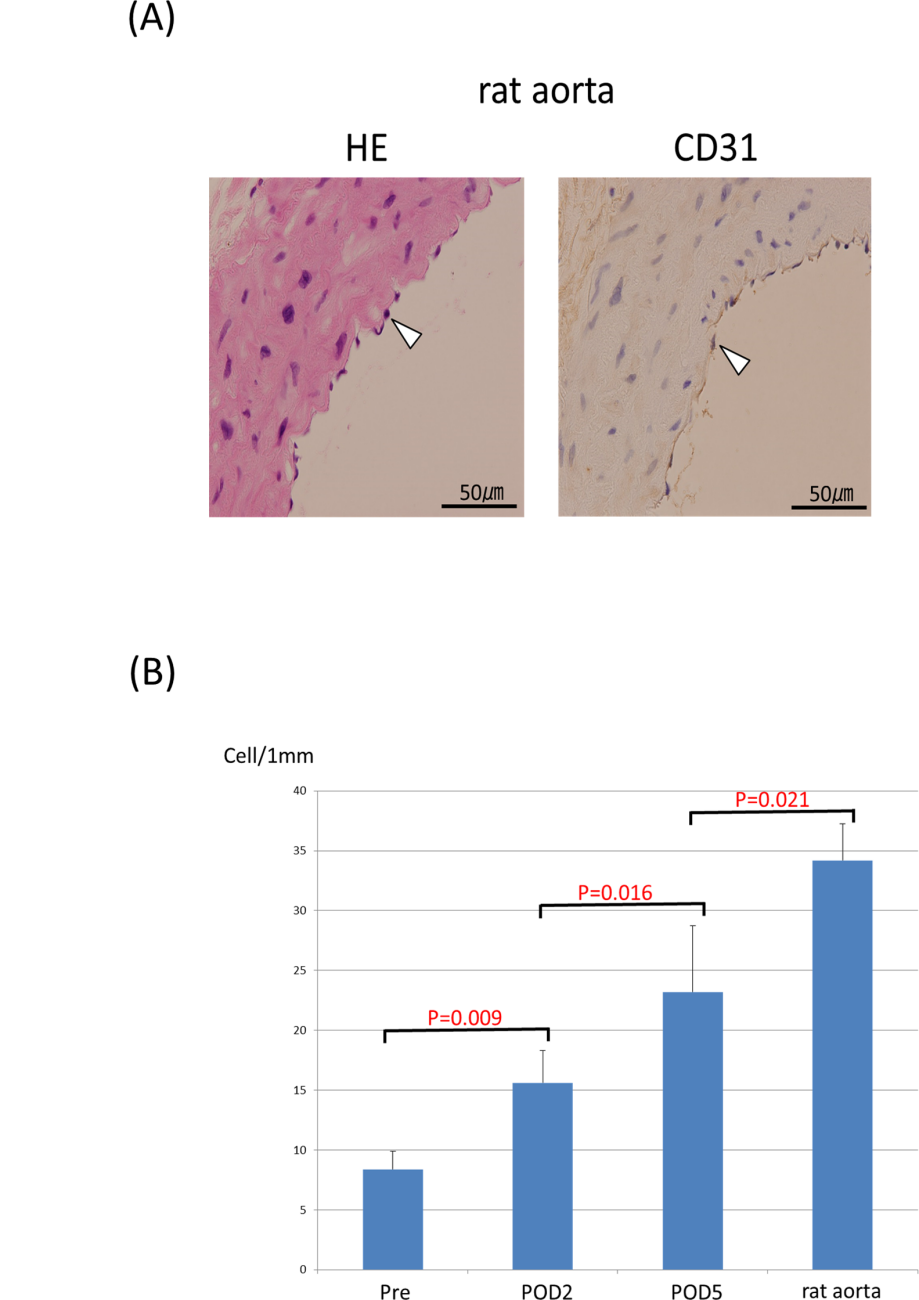
Histological examination of the luminal side of the vascular graft. At pre-implantation, the vascular endothelial cells distribute to the entire area of the graft. Conversely, after implantation, vWF, CD31 and CellTracker Red-positive endothelial cells are seen at the inner lumen of the vessel. Furthermore, the vascular endothelial cells cover the inner surface of the vessel more continuously on the fifth day than on the second day.

Although piercing of the spheroid resulted in making a hole immediately after the removal from the needle array, the pre-implantation vascular graft of the short axis cross-section showed complete closure of these holes (Fig 5). Masson’s trichrome staining revealed the extensive collagenous ECM as blue (Fig 5B). vWF and CD31-positive vascular endothelial cells were distributed to all parts of the tubular tissue (Fig 5C and 5D). After implantation, vWF, CD31 and CellTracker Red-positive endothelial cells were recognized at the inner side of the vessel. Furthermore, the vascular endothelial cells covered the inner surface of the vessel more continuously on the fifth day than on the second day (Fig 6). The number of endothelial cells in the tubular tissue, as well as in native rate aortae, was counted (Fig 7A). Significant increases in the number of endothelial cells were observed after implantation compared to the pre-implant tissue (Fig 7B).

### Remodeling of the tubular tissue after implantation

**Fig 8:**
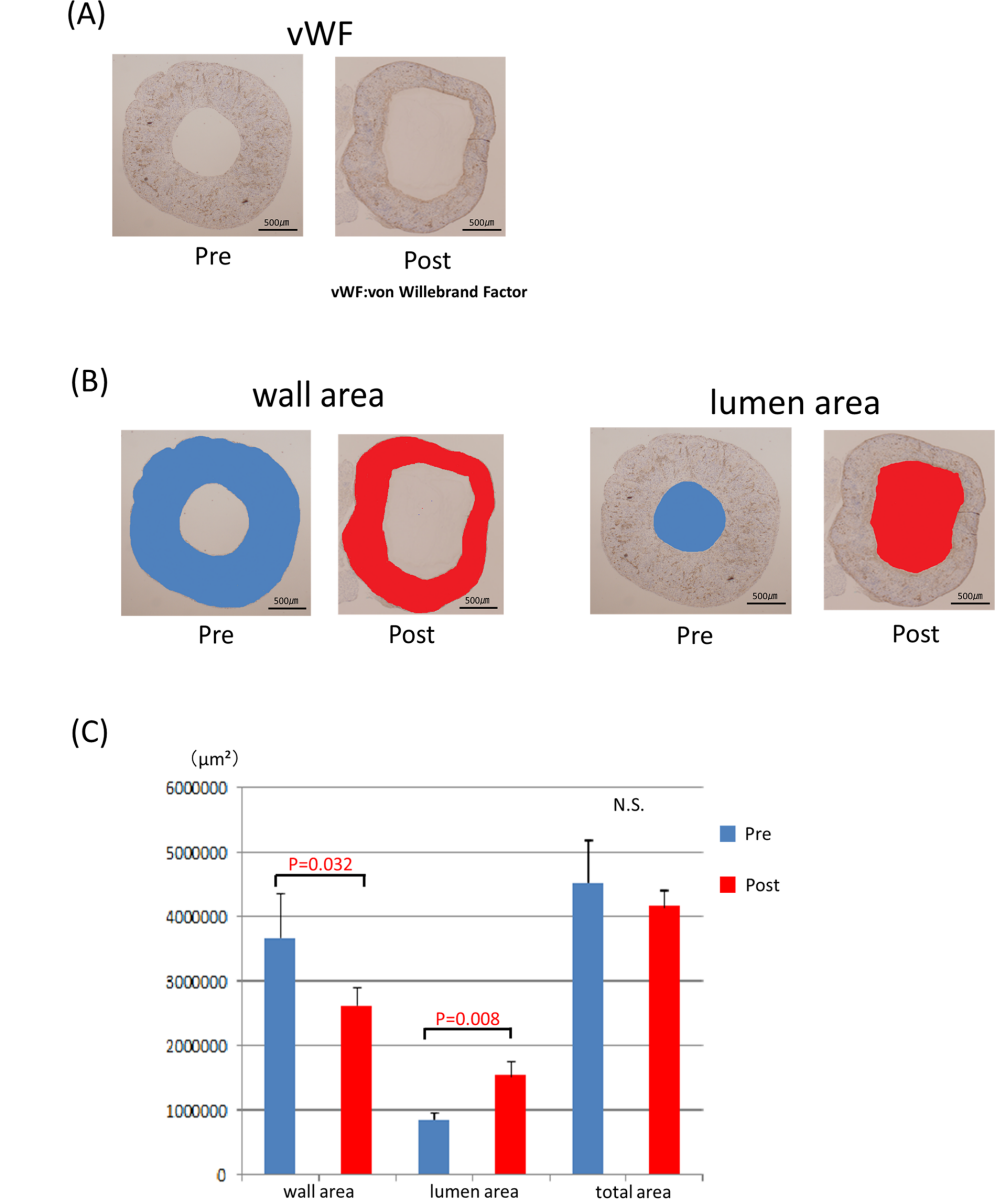
The rat aortae were stained with HE and CD31, respectively (A). The number of endothelial cells in the five rat aortae was counted and compared with those in the tubular tissues (B).

The lumen area and the wall area of the tubular tissue were measured before and five days after implantation (Fig 8A and 8B). The lumen area was enlarged (P = 0.032) and the wall area was decreased (P = 0.008) after implantation. The total area (the sum of the wall area and lumen area) showed no significant difference (Fig 8C).

## Discussion

We developed a novel method to create scaffold-free small caliber tubular tissue from MCSs using a “Bio-3D printer”-based system. We successfully implanted the tubular tissue into rats and showed that the inner luminal surface of the structure was covered with endothelial cells after implantation. Although several studies have attempted to use MCSs for tissue-engineered scaffold-free artificial vessels [14–16], to the best of our knowledge, this is the first report to show endothelial cells which cover the inner surface of the tubular tissue fabricated with a technology of regeneration and tissue-engineering when implanted *in vivo*.

Conventional vascular prostheses have significant limitations with regard to thrombosis, infection and biocompatibility, especially for small caliber blood conduits. Therefore, a wide range of research has been implemented for the development of small caliber vascular prosthesis using the approaches of regenerative medicine and tissue engineering [17, 18]. These approaches include the (1) the utilization of scaffolds comprising absorbent polymers or other substances [19–21], (2) the generation of tubular structures made of collagen with the usage of subdermally implanted silicon templates [22–25], (3) utilization of decellularized blood vessel tissue as a vascular graft [26], or (4) production of tubular tissues from self-assembled cells or tissue from the patient’s own tissue/organ without using a scaffold [27, 28].

L’Heureux and colleagues produced tubular tissue using the technology of cell sheet engineering [27]. They implanted the graft as a blood access for patients on chronic dialysis [29]. This method demonstrated significant potential, as the fact that no animal-derived or foreign substances were used, thereby avoiding the problems using foreign material which carries the issues of contamination, biotoxicity and allergy. The practical problem of this approach, however, was that it required nearly a 6-month period of *ex vivo* preparation before transplantation, because the formation of the cells from extracellular matrix production in the culture system required a significant amount of time. In addition, it requires several processes to make three components, that is living adventitia, decellularized internal membrane, and an endothelium. This makes this approach not only time-consuming, but also complicated.

As we have shown in the current study, a greater quantity of extracellular matrix was produced during a short period of time within the MCSs composed of endothelial cells, fibroblasts, and smooth muscle cells. The utilization of MCSs for tissue engineering takes advantage of the capacity of dissociated cells to aggregate through the mechanism of cell-cell attachment. This cell-cell attachment phenomenon has been known for over 100 years [30] and is a survival mechanism that allows cells to avoid anoikis, possibly by activating signals mediated by surface receptors and ligands which suppress the anoikis cascade. This phenomenon is preserved in almost all living organisms irrespective of their complexity [31].

MCSs can be generated from enzymatically and/or mechanically dissociated tissues of interest [32, 33], from cell lines [34–35], or from stem cells [36]. The MCS itself has several advantages for making scaffold-free 3-D biostructures.

First, the cells in an MCS show greater similarly to the cells in the native state than the cells in a monolayer culture [12]. The mechanism of this phenomenon is not clear, however, it may be due to the fact that an abundant extracellular matrix was produced in the MCS. Abundant collagen was additionally produced in the MCS, probably from the fibroblasts. The extracellular matrix may contribute to the reorganization of the cells within the MCS and also within the tubular structures after the MCSs fuse to each other. Although we could not demonstrate elastic fibers in the tubular structure, refinement of the process of fabrication of the tubular structure may enable one to generate elastic fibers in the biostructure.

Second, it is possible to control the total number of cells and the type of cells within the MCS. In the current study, the total number of cells was set to 2.5 x 10^4^ cells. The number was set due to the size of the tip of the robotically controlled nozzle. Smaller MCSs cause difficulty in handling with the nozzle, whereas larger MCSs would result in the thickening of the wall of the tube, which would cause a narrowing of the tubular structure. In terms of the cell source ratio, Marga et al. reported that they created tubular tissue from spheroids comprising a 5:14:1 mixture of human aortic endothelial cells (HAEC), HASMC and NHDFB [14]. We used a ratio of 4:1:5 of HUVEC, HASMC and NHDFB, respectively. We set the ratio of endothelial cells (HUVEC) relatively higher than described in previous reports, expecting to promote endothelialization of the tubular structure. Although we could demonstrate a good *in vivo* short-term function of the tube as a vascular graft, the optimal combination of cell sources and its ratio requires further investigation.

Finally, the approach using MCSs enabled us to include endothelial cells as a component of the MCS. A histopathological comparative observation demonstrated that the number of endothelial cells was smaller than that of the native aortae, however, the number increased as the implantation duration elongated, which may indicate that a mechanical factor (shear stress and/or pressure load) or contact with the blood flow was the trigger to arrange the endothelial cells. However, the exact mechanism of this phenomenon must be clarified. Of note, the endothelium covering the surface of the lumen did not originate from the donor. We clearly demonstrated from the staining that the endothelial layer originated from the cells mixed in the MCSs. The paracrine effect of the endothelium should be noted as well. Several proangiogenic factors, such as vascular endothelial growth factor (VEGF), may be produced within the MCSs, enabling the induction of either vascularization or angiogenesis within the MCSs or surrounding tissues after implantation [37–39]. Additionally, for the purpose of testing the clinical relevance and long-term patency, thrombosis, smooth muscle cell proliferation, and intimal hyperplasia should be thoroughly sought, in addition to the clarification of what constitutes “consistent and sufficient” endothelial cell coverage. Further studies are warranted in a larger animal model which enables long-term observation.

We utilized our originally developed “Bio-3D printer” to generate scaffold-free tubular tissue from MCSs composed of endothelial cells, fibroblasts and smooth muscle cells. The advantages of using this system are: (1) the size, shape and length of the tubular structure are designable and highly reproducible because of the computer controlled robotics system, (2) the graftable tubular structure can be created within a reasonable time (8 days), and (3) the constructed tissue does not contain foreign material, which may imply good resistance to infection. Our 3-D bio-printing system is highly manipulative for controlling the size and length of the tubular structure.

Regarding blood vessel wall remodeling, the pre- and post-transplantation data (after five days) showed the wall area to be significantly smaller post-transplantation (P = 0.032), and the lumen area had increased in size (P = 0.008). These results may imply the remodeling of the graft as the consequence of *in vivo* implantation, however, long-term observation is undeniably necessary to examine whether further dilatation may occur.

### Limitations

There are several limitations associated with the current study. First, we did not perform a control study with a venous graft due to the small size of the animal (rat). We, however, are in the process of comparing the vascular construct and a venous and/or PTFE graft in a porcine model. In this experiment, a detailed comparison should be possible with the currently available approach. Second, the observation period after transplantation was short. A long-term observation should be followed to examine the durability and antithrombotic features. The above-mentioned porcine model may also be useful in this long-term observation. In addition, the underlying mechanisms of endothelialization and migration of the cells in the tubular tissue have not yet been identified, such as shear stress, stimulation of the luminal surface by circulating substances in the blood, or by stimulus from surrounding connective tissue. With regard to elucidating the antithrombogenicity of the endothelial cells, we performed additional staining with anti-thrombomodulin and anti-tissue factor. However, positive staining was not observed along the luminal surface of the tissue (data not shown). Further studies are warranted to demonstrate the biological mechanism of the cell location and function within the tubular tissue, as well as to examine the long-term results of the implanted tubular structure. Finally, examination of mechanical property of the vascular tissue is needed. We are currently testing the mechanical property of the vascular construct, and at this moment the construct is durable over 939 mN, which is about half of the value of the native vessel, according to a tension test apparatus. For clinical application, mechanical property comparable to native vessel is definitely required, such as the measurement of the burst pressure (expressed as mmHg) of the tubular tissue.

## Conclusion

The scaffold-free tubular tissues made of MCS using a Bio-3D printer underwent remodeling and endothelialization. Further studies are warranted to elucidate the underlying mechanism of endothelialization and its function, as well as the long-term results.

## Supporting Information

S1 Video The Bio-3D Printer constructing scaffold-free tubular tissue. (WMV)
