# Correction: Scaffold-Free Tubular Tissues Created by a Bio-3D Printer Undergo Remodeling and Endothelialization when Implanted in Rat Aortae

**DOI:** 10.1371/journal.pone.0145971

**Published:** 2015-12-29

**Authors:** Manabu Itoh, Koichi Nakayama, Ryo Noguchi, Keiji Kamohara, Kojirou Furukawa, Kazuyoshi Uchihashi, Shuji Toda, Jun-ichi Oyama, Koichi Node, Shigeki Morita

The captions for Figs 6, 7 and 8 are incorrectly switched. The caption that appears with Fig 6 should be with Fig 8; the caption that appears with Fig 7 should be with Fig 6; the caption that appears with Fig 8 should be with Fig 7. Please view Figs 6, 7 and 8 with the correct captions here.

**Fig 6:**
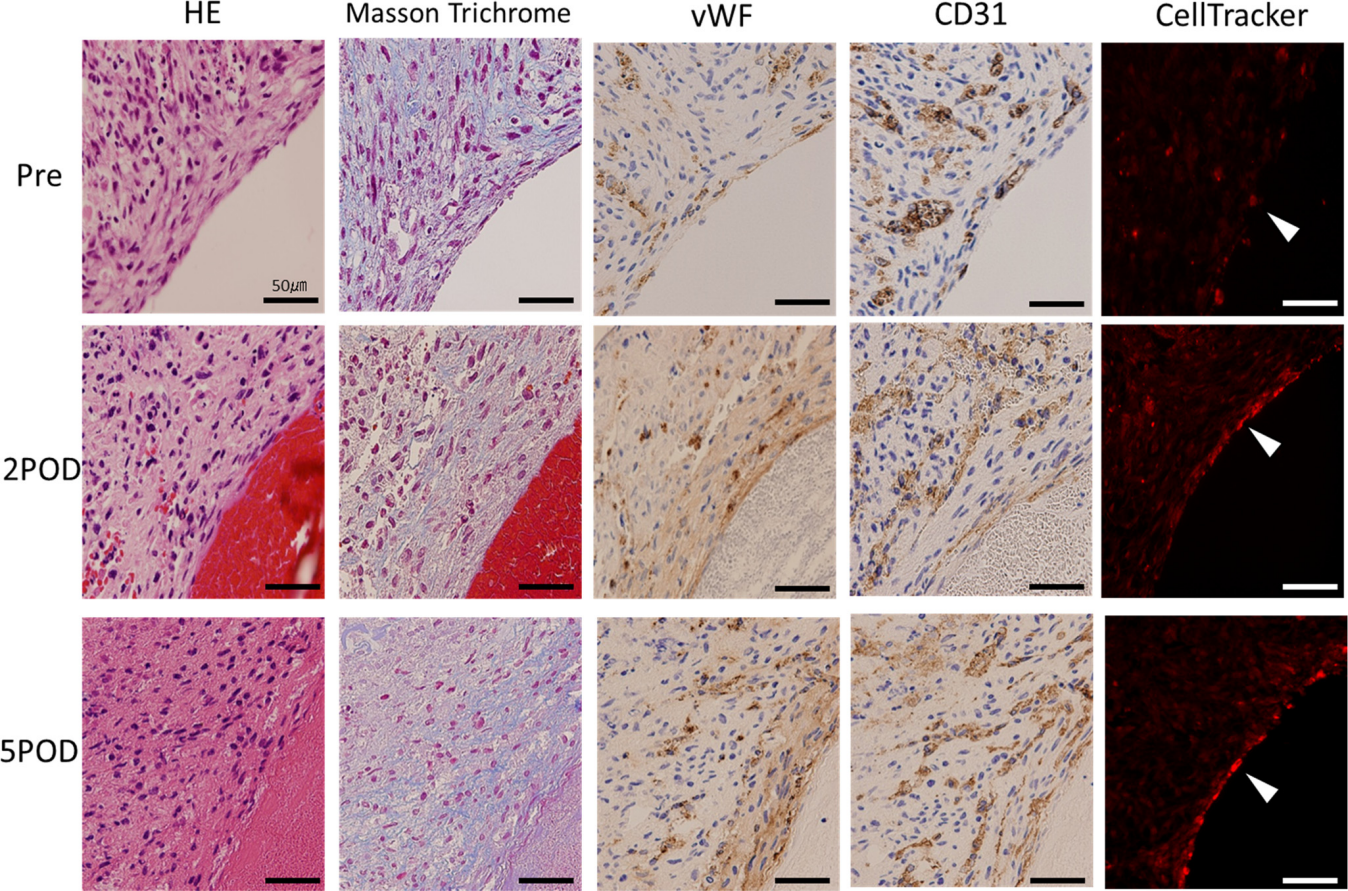
Histological examination of the luminal side of the vascular graft. At pre-implantation, the vascular endothelial cells distribute to the entire area of the graft. Conversely, after implantation, vWF, CD31 and CellTracker Red-positive endothelial cells are seen at the inner lumen of the vessel. Furthermore, the vascular endothelial cells cover the inner surface of the vessel more continuously on the fifth day than on the second day.

**Fig 7:**
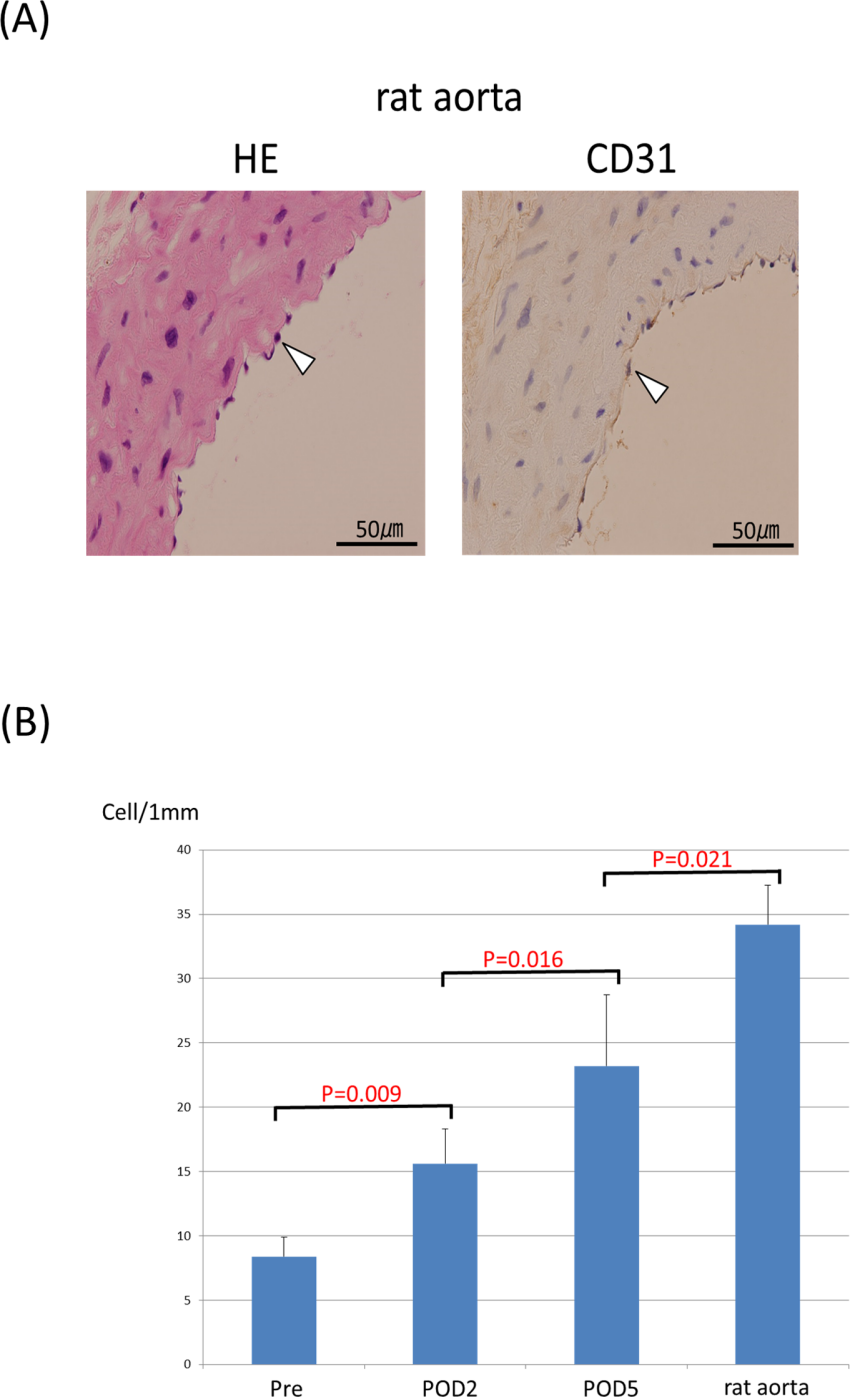
The rat aortae were stained with HE and CD31, respectively (A). The number of endothelial cells in the five rat aortae was counted and compared with those in the tubular tissues (B).

**Fig 8:**
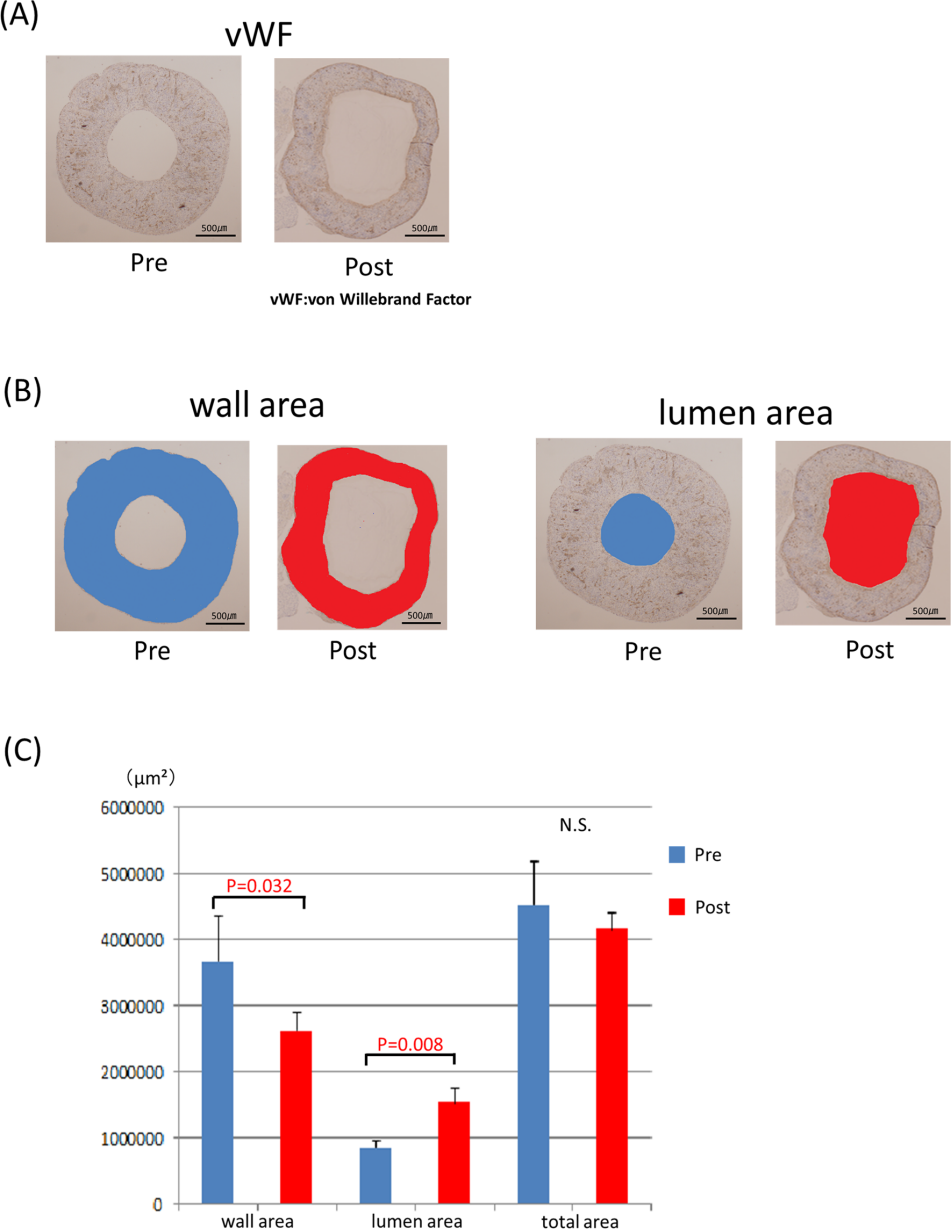
Remodeling of the blood vessel (Post-implantation). The graft of post-implantation is patent and remodeled (A). The wall area and lumen area pre-implantation are shown in blue, post-implantation in red (B). The lumen area is enlarged (P = 0.032) and the wall area is decreased (P = 0.008) after implantation. The total wall area and lumen area shows no significant difference (C).
